# Anatomo-Clinical Aspects of Retinoblastoma: A Series of 144 Cases

**DOI:** 10.7759/cureus.25422

**Published:** 2022-05-27

**Authors:** Fidélia Nihad Da Silva, Lafia Xavier Kora, Tania Elongo, Asmaa El kebir, Loubna El maaloum, Nisrine Bennani-Guebessi, Bouchra Allali, Asmaa El kettani, Mehdi Karkouri

**Affiliations:** 1 Anatomical Pathology, Ibn Rochd University Hospital Center, Casablanca, MAR; 2 Pediatric Ophthalmology, Ibn Rochd University Hospital Center, Casablanca, MAR; 3 Pediatric Ophthalmology, Ibn Rochd University Hospital Center, Casblanca, MAR

**Keywords:** pediatrics, strabismus, leukocoria, histopronostic factors, retinoblastoma

## Abstract

Retinoblastoma (RB) is the most common intraocular primary malignancy for infants and young children. The tumor is bilateral in 40% of cases and unilateral in 60% of cases. The hereditary form is due to a germinal mutation in the RB1 tumor suppressor gene. In developed countries, patients treated for RB have excellent survival, but unfortunately in developing countries delays in diagnosis and lack of human and financial resources are responsible for deaths.

We conducted a retrospective study of 144 cases of RB in order to evaluate the clinico-pathological aspect of RB for the national reference center of RB in Morocco. Our study highlighted the indispensable collaboration between the clinician and the pathologist. Besides the diagnostic confirmation, the anatomopathological study gives us information on histopronostic risk factors to guide the treatment.

## Introduction

Retinoblastoma (RB) is the most common intraocular primary tumor in infants. It is a rare tumor with an estimated occurrence of one out of 20,000 births [1]. The tumor is bilateral in 60% of cases and unilateral in 40% of cases. RB is caused by a mutation of the RB1 gene located on 13q14.

The two most frequent clinical signs are leukocoria and strabismus. The prognosis is good in developed countries, due to early diagnosis and management, in comparison to underdeveloped countries where the prognosis is poor due to late diagnosis. The general histopathological feature of RB is the presence of small rounded blue monomorphic cells that may or may not form rosettes depending on the degree of differentiation [2]. Other commonly observed histopathological aspects include necrosis, calcification, and also tumor extension to different anatomical parts of the eye.

It is a diagnostic and therapeutic emergency to save the functional and vital prognosis hence the interest in an early quality diagnosis and management based on histoprognostic elements. The objective is to describe the clinicopathological findings of RB and to correlate the histological criteria with progression.

## Materials and methods

This is a retrospective and descriptive study of all infants with RB who underwent enucleation over a period of nine years and 11 months, from January 2010 to December 2020, followed in the Pediatric Ophthalmology Department at the Ibn Rochd University Hospital Center of Casablanca (Morocco). We analyzed the following parameters: patient's age, sex, consanguinity, laterality, clinical aspects revealing signs, consultation delay, international RB classification, the number of courses of chemo-reduction received before enucleation, macroscopic examination (tumor, size), histological type according to the World Health Organization (WHO) classification, histopronostic factors, evolution.

The diagnosis is made through an ophthalmologic examination of the child under general anesthesia. The anterior segment and the fundus of the child's eye are examined after pupillary dilation to assess the condition of the posterior pole, the mid-periphery, and the ora serrata. A diagram of the tumor(s) is made to indicate its size, and its location in relation to the macula, papilla, equator, and ora serrata. The presence and extent of retinal detachment, intravitreal or subretinal swelling. The shape of the RB is assessed with the international classification of RB.

An ocular ultrasound often helps especially in case of differential diagnosis with Coats’ disease. In RB, the ultrasound often finds calcifications. Neuroimaging (cranio-orbital scan, magnetic resonance imaging) is performed to evaluate the loco-regional extension. In extraocular forms, a general extension assessment is performed immediately.

Neoadjuvant chemotherapy is used to facilitate enucleation or to reduce the tumor in order to make conservative treatment possible. Three types of situations can be distinguished. First, when the goal is to facilitate enucleation, the treatment consists of a combination of carboplatin (200 mg/m^2^ per day for three days), etoposide (100 mg/m^2^ per day for three days), and vincristine (1.5 mg/m^2^ for one day). One to two repeated courses at three-week intervals are usually sufficient. Second, when the goal is to reduce the size of the tumor and make it accessible for focal treatment, the cure includes the combination of carboplatin (560 mg/m^2^ per day for one day) and vincristine (1.5 mg/m^2^ per day for one day). Usually, one to six courses are given at three-week intervals. Third, when post-enucleation chemotherapy is performed because of the presence of positive histopronostic factors. It combines CADO (vincristine, cyclophosphamide, doxorubicin) and carboplatin-etoposide courses. In general, four to six courses of treatment are given, depending on whether there is a medium or high histoprognostic factor.

Our study was only interested in eyes that have been enucleated. Enucleation in RB surgery must avoid any scleral effraction and ensure that the optic nerve is cut as far away from the eyeball as possible. Enucleation is indicated in extraocular RB after one or two courses of chemoreduction, or in advanced stages of RB. It is also indicated in bilateral RB after chemoreduction on the more affected eye or exceptionally on both eyes when there is no longer a visual prognosis.

Once enucleated, it was sent to the anatomy and pathological cytology laboratory in a fresh state where it was treated according to a very precise protocol. Indeed, after an external description and measurements of the eyeball, the limit of the optic nerve is removed separately. It is then fixed in formalin for at least 48 hours, with an adequate volume, rinsed with running water for 16 hours before being placed in 60% alcohol for 30 minutes. After removal of the resection limit of the optic nerve in a cassette, and orientation of the specimen, the eyeball is sectioned, followed by an internal description of the tumor with the different structures, and lastly a complete removal in 6 blocks comprising: the upper and lower cap, the upper and lower medial collar, and the upper and lower middle collar of the eyeball. Finally, a kerosene embedding was performed followed by a hematoxylin and eosin staining. An immunohistochemistry study was rarely requested and necessary.

## Results

Clinical data

**Figure 1:**
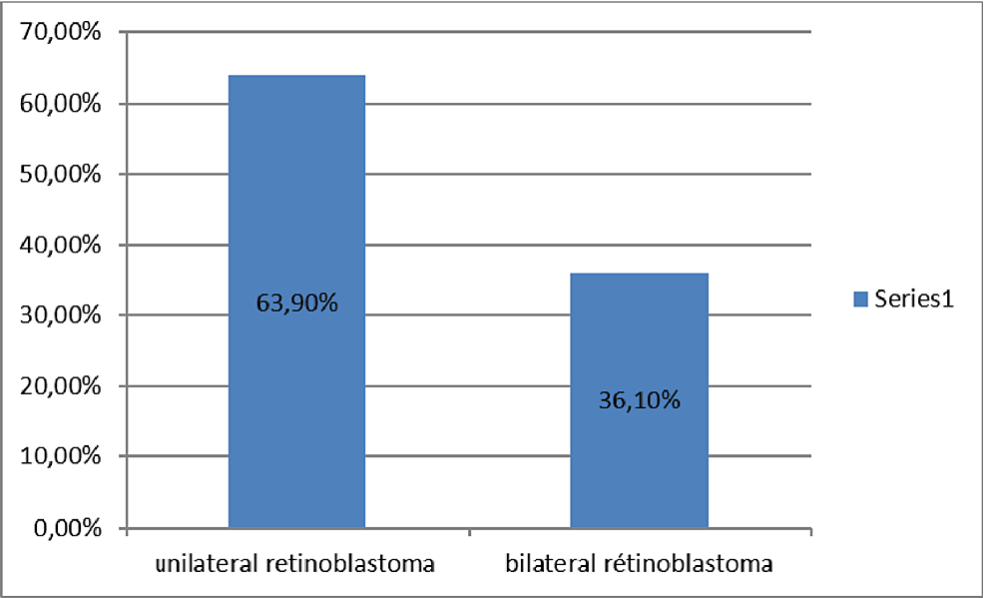
Graph showing the laterality of retinoblastoma

Our study included 144 patients, 77 girls and 67 boys. The sex ratio (boy/girl) = 0.87. The mean age of the patients in our study was 26.06 ± 19.29 months. The median age was 24 months. 95.8% of the patients in our series were less than five years old and 4.2% of the patients in our series were older than five years. Consanguinity was found in 26 patients, which represents 18.1% of patients. In our series, RB was unilateral in 63.9% (92 cases) and bilateral in 36.1% (52 cases) (Figure 1).

**Figure 2:**
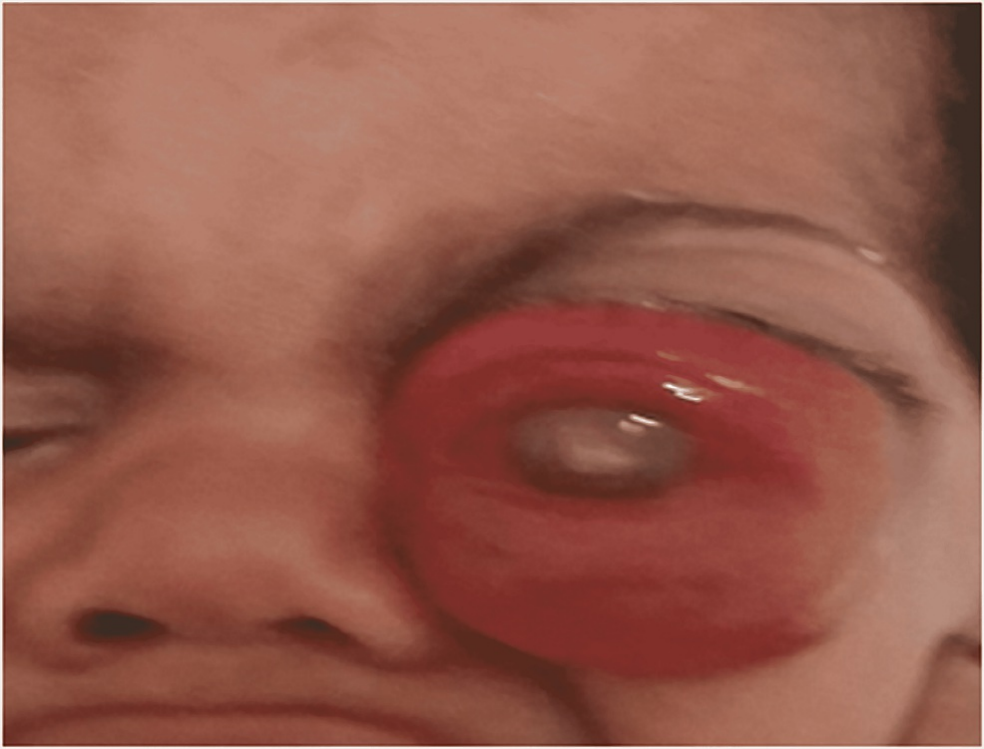
Photograph of a left exophthalmos revealing a retinoblastoma

**Figure 3:**
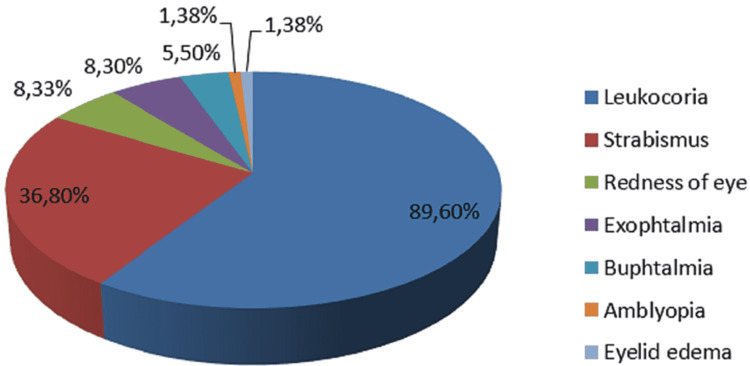
Diagram representing the reasons for consultation

In the unilateral forms, we counted 47 cases in the left eye (51.09%) and 45 cases in the right eye (48.91%). Regarding the reasons for consultation, the most frequent sign was leukocoria. Leukocoria was found in 129 patients which represents 89.6% of our cases. The second most frequent sign was strabismus found in 53 patients (36.80% of cases). In the third position, redness of the eye and exophthalmia were found in 12 patients (8.33% of cases) (Figure 2). In the fifth/fourth position, buphthalmia was found in 8 patients (5.5% of cases). Amblyopia and eyelid edema was found in two patients (1.38% of cases) (Figure 3).

The mean time of diagnosis was 4.08 ± 5.7 months with extremes ranging from 0 to 52 months. The median time of diagnosis was two months with an interquartile range of one to 5.5 months. According to the international classification of RB, 11 eyes were classified as stage A (5.61%), 13 eyes were classified as stage B (6.63%), 13 eyes were classified as stage C (6.63%), 34 eyes were classified as stage D (17.35%) and 125 eyes were classified as stage E (63.78%).

In our study, 86 patients (59.72%) received chemoreduction treatments before enucleation. The average number of chemoreduction treatments was 2.30 with extremes ranging from one to six. Regarding the evolution of our patients. Eight patients were lost to follow-up (5.5% of patients). A total of 123 patients or 85.41% of patients were alive, and 13, or 9.02% of patients has died. In relation to death, six patients died of metastasis, five patients died of medullary aplasia syndrome, one patient died of a respiratory infection, and one more of a convulsion of undetermined cause.

Macroscopic data

**Figure 4:**
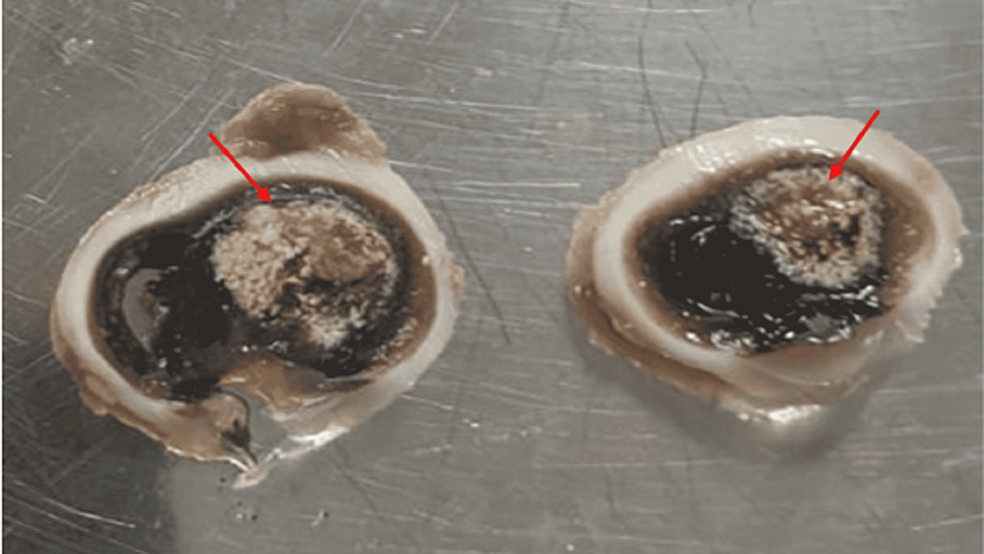
Macroscopic examination of an enucleation specimen showing a friable whitish tumor occupying half the vitreous

On macroscopic examination of the enucleation specimens, a whitish tumor (Figure 4) was described in 94.1% of cases, varying in size from 0.5 to 2.7 cm in the long axis, with an average of 1.5 cm, and retinal detachment was observed in 16 cases (11.11%).

Microscopic data

**Figure 5:**
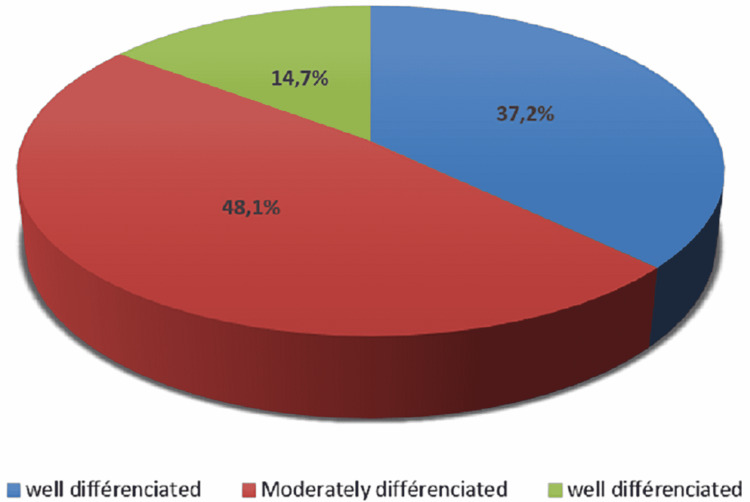
Distribution of histological subtypes

**Figure 6:**
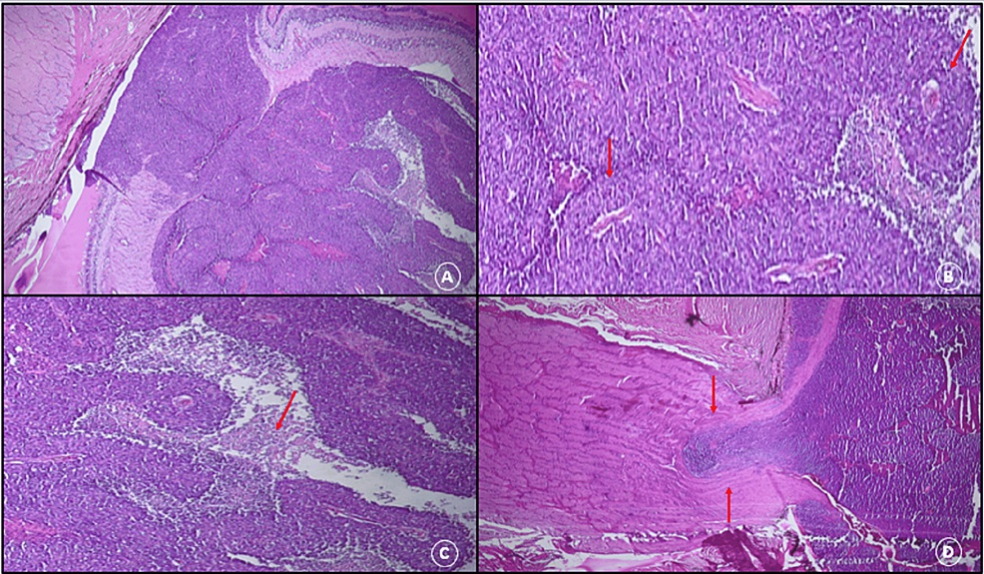
Microscopic appearance of a retinoblastoma. (A) Viable small blue cell tumor proliferation arranged in diffuse sheets with pseudo-rosettes (hematoxylin-eosin, original magnification ×40). (B) Small round viable cells forming pseudo-rosettes (HE ×200). (C) Necrotic remnants after chemotherapy (HE×100). (D) Post-laminar infiltration of the optic nerve (HE ×40).

Histological examination showed in 89.6% of cases (129 cases) a viable malignant small round cell tumor with three distinct forms of RB differentiation (Figure 5). A well-differentiated form in 19 cases (14.7%), a predominantly moderately differentiated form with pseudo-rosettes in 62 cases (48.0%), and a poorly differentiated form in 48 cases (37.2%) (Figures 6A, 6B). Associated foci of calcification and necrosis were found in 81 cases (62.7%) and 100 cases (77.5%) respectively (Figure 6C). In all the cases of dying patients, nine eyes (69,3%) had a poorly differentiated RB and only four eyes (30,7%) had a moderately differentiated RB.

**Figure 7:**
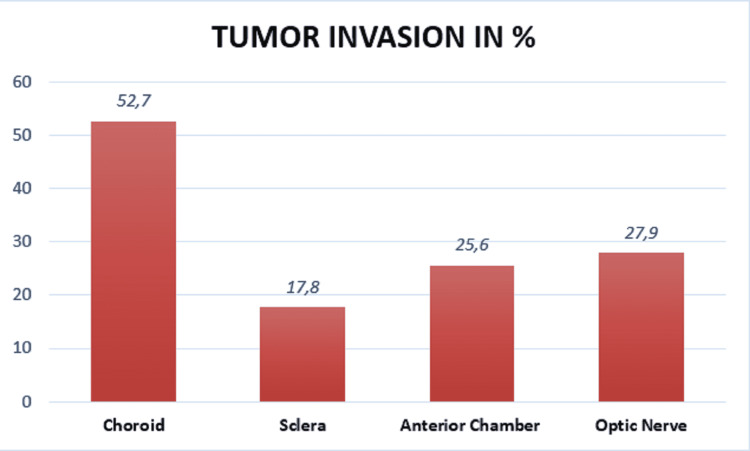
Tumor extension of retinoblastoma.

Tumor extension into the choroid (minimal and massive) was found in 68 cases (52.7%) with scleral involvement in 23 cases (17.8%) (Figure 7). The anterior chamber was invaded in 33 cases (25.6%). Invasion of the optic nerve was present in 27.9% (n = 36). This invasion was pre-laminar in 19 cases (14.7%), retro-laminar (Figure 6D) in 13 cases (10.1%) and was present at the level of the sectional trench in four cases (3.1%). Among the 13 patients who died, seven patients (53.8%) had retro-laminar optic nerve invasion, and four patients (30.7%) had an invasion of the sectional trench.

In 15 cases, there were mainly post-chemotherapy changes without viable cells. Mostly 80% (12 cases) were large areas of necrosis mixed with calcifications, and more rarely in three cases (20%), diffuse fibrosis was found.

## Discussion

Epidemiology

RB is the most common intraocular primary tumor seen in children [3]. It is a serious pathology that is life-threatening and visually damaging, with important socioeconomic consequences, hence there is a need for early diagnosis and multidisciplinary management.

The tumor is unilateral in 60% of cases and bilateral in 40% [3]. These data are consistent with our study, as the bilateral form represented 36.1% of cases and the unilateral form represented 63.9% of cases. RB is caused by a mutation in the RB1 tumor suppressor gene located on 13p14. In familial forms, the patient carries a mutation on all his cells and a mutation on the other chromosome is sufficient for the tumor to develop. Bilateral forms are usually multifocal. In unilateral forms, both mutations must develop in the same cell. In unilateral tumors, there is a risk of bilateralization which requires lifelong ophthalmological surveillance [4].

According to Shields, more than 90% of RB cases are diagnosed before the age of five years [5], which is consistent with our study, as 95.8% of the patients in our series were younger than five years old. In our study, we noted a slight female predominance with a sex ratio of 0.87 whereas the literature does not find a gender predominance in the occurrence of RB. This discrepancy with the literature could be explained by the fact that our series was small.

Clinical signs

The most common signs are leukocoria and strabismus [6]. Strabismus is the earliest sign of macular damage. Leukocoria is a late sign that is not always visible at first. The role of the family, pediatricians, and general practitioners is very important to report the leukocoria.

There are other signs of revelation such as buphthalmia, pseudo-cellulitis, hyphema, and iritis rubeosis. Our results are consistent with those of the literature, with a predominance of leukocoria (89.6%), followed by strabismus (36.80%). In the series by Chebbi et al., leukocoria was found in 58.5% of cases and strabismus in 16.5% of cases [6].

The mean time of diagnosis in our series was 4.08 months which is consistent with the series of Chebbi et al in which the meantime to diagnosis was 4.8 months. In our series, at the time of diagnosis 81.13% of the eyes were diagnosed at an advanced stage of RB versus 73.3% in the series of Chebbi et al. The delays in diagnosis associated with the difficulties of regular access to the only specialized center in the management of RB leads to an evolution of the tumor requiring recourse to enucleation to preserve the vital prognosis.

In developed countries, the five-year survival rate is over 90%. This has been found in many studies [7]. The overall survival rate at five years was 87.5% in the series by Bouguila et al. in 1999 [8]. These data from the literature correspond to our series where the overall survival rate at five years is 85.41%. The factors that worsen the prognosis are the size of the tumor and the extra-retinal extension with the invasion of the choroid, the sclera, or the optic nerve [9]. Thirteen or 9.02% of the patients in our series died. Six patients died in the context of metastases and five patients died in the context of a bone marrow aplasia syndrome. Indeed, the natural evolution of RB is toward complications and locoregional and metastatic extension resulting in death. However, the literature reports spontaneous tumor regression in 2% of cases [3].

With the improvement in survival, the number of cured patients is increasing. These patients in adulthood need genetic counseling [10]. Molecular diagnosis, when it identifies the mutation, makes it possible to predict recurrence in siblings or transmission to offspring through the prenatal or neonatal diagnosis of carriers.

Macroscopy

Pathological examination is the key examination for tumor typing and for determining histopronostic features. The International Retinoblastoma Staging Working Group (IRSWG), consisting of 58 participants from 24 countries on four continents, organized a series of web-based meetings in 2009 to discuss staging and tissue handling guidelines to reach a consensus for adequate treatment, establish definitions of histopathological risk factors and report enucleated eyes with RB [11]. Two basic techniques have been proposed to safely retrieve the tumor without extensive tumor contamination of ocular structures [11]. In our study, following the example of France and Tunisia [6,12,13], six cassettes were performed systematically with full inclusion of the enucleation specimen. In India, five blocks were performed. The histological examination of the enucleation specimen must be carried out by an experienced pathologist, as it conditions the postoperative therapeutic indications. The mean tumor size in our series was 1.5 cm, which is similar to a study in India where RB ranged from 0.4 to 5 cm with a mean of 1.5 cm [12,13].

Histology

Histologically, it is a small round cell blue tumor composed of primitive neuroblastic cells that appear blue with highly basophilic nuclei and a little cytoplasm. The differentiation of RB is characterized by the presence of Flexner-Wintersteiner rosettes, florets, and Homer-Wright rosettes which are non-specific [2,14]. The moderately differentiated form of RB was the most common in 48% of our series. Owoeye et al. [15] reported 17.4% of well-differentiated cases, which is almost similar to the 14.7% in our study. This low representation of the well-differentiated form has been observed in other settings, particularly in developing countries, with sometimes lower rates such as 6.7% reported by Nyaywa and associates [2,16] in Zambia. In contrast, in developed countries, well-differentiated forms were the most common, with a higher proportion as reported by Eagle and associates [17] in the USA (41%). This notable difference in tumor differentiation in RB between developing and developed countries has been associated with better health care-seeking behavior, availability of pediatricians and ophthalmologists in lower health care settings, and reasonable socioeconomic status (SES) in developed countries, all resulting in diagnosis and treatment [2,18]. Differentiation in RB does not appear to have a prognostic role.

The histopronostic factors to look for are choroidal invasion either minor (<3 mm in the largest dimension) or major (>3 mm in the largest dimension of a single lesion or sum of all areas of choroidal invasion), tumor extension to the optic nerve, extraocular extension and anterior chamber invasion [2,19]. Depending on their degree of importance, they are usually considered to be a major risk for metastasis and therefore poor prognosis [2]. The proportion of cases with the choroidal invasion of 52.7% in this series was higher than 24.4% in a Tunisian case series [6,12], 27.3%, and 18% reported by Yahaya, Uganda, and Reddy and Anusya, Malaysia, respectively [2]. The difference could be due to the different sample sizes between the series. In the series by Darwich et al. [20], it was reported that patients with choroidal invasion were more likely to develop systemic metastases compared to those with or without optic nerve invasion. The Choroidal invasion has also been shown to correlate with molecular prognostic markers such as TP53, in contrast to other high-risk factors. Optic nerve invasion appears to predict metastasis and mortality, especially when the extent of invasion is taken into account [2,21]. Optic nerve involvement in our series was found in 27.9%; a rate similar to the 25% reported by Chebbi et al. in Tunisia [6], or the 26.5% reported by Dial et al. in Dakar [12]. However, its risk to the results seems to be limited to the involvement beyond the sieve blade and the surgical margin of the optic nerve [2,18]. Which is consistent with our results. Both optic nerve and choroidal invasion have shown strong and independent prediction of metastasis in most studies.

Thus, high-risk RB has been defined as the presence of tumor seeds in the anterior chamber, iris/ciliary body infiltration, massive (≥3 mm) choroidal invasion, post-laminar optic nerve infiltration, optic nerve resection limit, combined pre-laminar/laminar optic nerve infiltration and minor choroidal invasion, or scleral/extra scleral tumor infiltration [17,19].

## Conclusions

RB is one of the most common cancers in children under five years of age. It is a disabling disease with a prevalence but also mortality and morbidity due to this disease are still high in developing countries including Morocco. Hence, the need for early detection of the disease with an awareness of parents as soon as the first clinical signs appear. Treatment is fairly well codified, from surgery to chemotherapy and depending on imaging and histopronostic factors.
